# RGS10 Reduces Lethal Influenza Infection and Associated Lung Inflammation in Mice

**DOI:** 10.3389/fimmu.2021.772288

**Published:** 2021-11-29

**Authors:** Faris Almutairi, Demba Sarr, Samantha L. Tucker, Kayla Fantone, Jae-Kyung Lee, Balázs Rada

**Affiliations:** ^1^ Department of Infectious Diseases, College of Veterinary Medicine, University of Georgia, Athens, GA, United States; ^2^ Department of Pharmaceutical and Biomedical Sciences, College of Pharmacy, University of Georgia, Athens, GA, United States; ^3^ Department of Physiology and Pharmacology, College of Veterinary Medicine, University of Georgia, Athens, GA, United States

**Keywords:** regulator of G-protein signaling (RGS)10, G-proteins, influenza A virus, monocytes, neutrophils, lung inflammation, cytokines, chemokines

## Abstract

Seasonal influenza epidemics represent a significant global health threat. The exacerbated immune response triggered by respiratory influenza virus infection causes severe pulmonary damage and contributes to substantial morbidity and mortality. Regulator of G-protein signaling 10 (RGS10) belongs to the RGS protein family that act as GTPase activating proteins for heterotrimeric G proteins to terminate signaling pathways downstream of G protein-coupled receptors. While RGS10 is highly expressed in immune cells, in particular monocytes and macrophages, where it has strong anti-inflammatory effects, its physiological role in the respiratory immune system has not been explored yet. Here, we show that Rgs10 negatively modulates lung immune and inflammatory responses associated with severe influenza H1N1 virus respiratory infection in a mouse model. In response to influenza A virus challenge, mice lacking RGS10 experience enhanced weight loss and lung viral titers, higher mortality and significantly faster disease onset. Deficiency of *Rgs10* upregulates the levels of several proinflammatory cytokines and chemokines and increases myeloid leukocyte accumulation in the infected lung, markedly neutrophils, monocytes, and inflammatory monocytes, which is associated with more pronounced lung damage. Consistent with this, influenza-infected *Rgs10*-deficent lungs contain more neutrophil extracellular traps and exhibit higher neutrophil elastase activities than wild-type lungs. Overall, these findings propose a novel*, in vivo* role for RGS10 in the respiratory immune system controlling myeloid leukocyte infiltration, viral clearance and associated clinical symptoms following lethal influenza challenge. RGS10 also holds promise as a new, potential therapeutic target for respiratory infections.

## Introduction

Seasonal influenza epidemics, with influenza A virus (IAV) being the most prevalent species, are estimated to result in 3-5 million severe illnesses and 300,000-650,000 deaths globally each year (1). Despite the COVID-19 pandemic, these influenza epidemics continue to represent a significant financial burden and a major global health threat. Seasonal viruses naturally mutate and consistently circulate (2), generating strains resistant to existing vaccines or antiviral therapies (3–5).

In response to IAV infection and subsequent replication, infected airway epithelial cells and activated lung-resident immune cells release inflammatory cytokines and chemokines to initiate a robust influx of innate immune cells, such as neutrophils, monocytes and macrophages into the lung (6). While the initial responses of neutrophils and macrophages are essential for IAV clearance, dysregulated and persistent inflammatory cell recruitment mediating uncontrolled inflammation leads to pulmonary damage and increased morbidity and mortality (7, 8). Therefore, identifying key regulators controlling excessive immune cell recruitment and the subsequent proinflammatory cytokine storm bears significance in the development of strategies for the treatment of influenza infections.

Regulator of G-protein signaling (RGS) proteins represent a superfamily of proteins defined by the presence of RGS domain, which is known to bind and deactivate heterotrimeric G-protein subunits (9, 10). Classically, RGS proteins modulate the magnitude and duration of G protein-coupled receptor (GPCR) signaling through heterotrimeric G-protein inactivation by accelerating the intrinsic GTPase activity of Gα-subunits to enhance the hydrolysis of the active guanosine triphosphate (GTP)-bound Gα subunits to inactive guanosine diphosphate (GDP)-bound Gα proteins (9–11). Because of the involvement of GPCRs and G-protein signaling in diverse systems, RGS proteins have emerged to mediate essential roles in regulation of physiological and pathological processes (12).

Among RGS proteins, RGS10, a member of the D/R12 subfamily, is one of the smallest RGS proteins that lacks structural domains and functional motifs outside of the RGS domain (13). RGS10 selectively functions as a GTPase activating protein (GAP) for Gαi family of G-proteins (11, 14, 15), and is highly expressed in the central nervous system (CNS) and immune system (16). Within the immune system, RGS10 has high levels of expression in spleen, lymph nodes, and the bone marrow as well as subsets of leukocytes including monocytes (17), tissue resident and recruited macrophages (18–20), dendritic cells (21), and T lymphocytes (22).

Independent of its GAP function, RGS10 demonstrates profound, *in vitro*, anti-inflammatory effect by regulating macrophages activation. Strikingly, loss of RGS10 in macrophages results in amplification of NF-*κ*B transcriptional activity and the generation of pro-inflammatory mediators, such as tumor necrosis factor-alpha (TNF-α), interleukins, and cyclooxygenase-2 (COX-2)-mediated prostaglandin E2 (PGE2), and inducible nitric oxide synthase (iNOS) upon toll-like receptor 4 (TLR4) activation (18–20, 23, 24). Further, following macrophage activation, RGS10 acts as a key factor in the regulation of macrophage polarization by suppressing classical M1 activation and promoting alternative M2 activation (19). In addition to macrophages, RGS10 regulates chemotaxis and adhesion of T cells in response to chemokine signals (22). While these observations propose a role of RGS10 in controlling immune cell activation, it remains unknow whether RGS10 plays a role in limiting exacerbated immune responses in respiratory infections including lethal influenza A viral infection.

In this study, our goal is to determine the *in vivo* biological role of RGS10 in IAV infection. Owing to the high expression of RGS10 in immune cells and its regulatory role in inflammatory responses, we predict that RGS10 has a physiological function in respiratory IAV infection. We hypothesize that *Rgs10* deficiency results in a prolonged and exacerbated inflammatory response associated with more severe clinical outcomes in a mouse model of influenza lung infection. Our results show that *Rgs10*-deficient mice are more susceptible to IAV infection than control mice. Significantly higher mortality, morbidity, lung viral loads and inflammation were observed in infected *Rgs10*-deficient mice compared to infected wild-type mice indicating a novel, beneficial role of RGS10 in the immune system, specifically respiratory antiviral responses.

## Materials and Methods

### Mice

Generation of *Rgs10*-deficient mice (*Rgs10-/-*) was described previously (18). Briefly, mice were backcrossed on a C57BL/6 genetic background for 10 generations. All *Rgs10-/-* mice and their wild-type (WT) C57BL/6 littermates were maintained in specific, pathogen-free animal facility, bred and housed in individually ventilated cages, with free access to food and water on campus in the Coverdell Vivarium at the University of Georgia. Pups were weaned from the mothers at a standard 21 days of age unless they appeared unable to support themselves, in which case they were weaned at 28 days. Genotyped animals were age- and sex-matched, randomly allocated to experimental groups, and randomly assessed. For identification purposes, standard ear-tagging was done. Animals were euthanized with CO_2_ and subsequent cervical dislocation when appropriate. No experimental drugs were used in this study. The following PCR primers were used to genotype mice:

*Rgs10* forward: 5’-CCACGAGGAAGTGAAGTGAAAGCTTT-3’,

*Rgs10* reverse: 5’-AGTCAGTTCTGAGTGTGTGAAAGTGC-3’, and

*LTR2*: 5’ AAATGGCGTTACTTAAGCTAGCTTGC-3.

The expected product size for the WT band is 400 bp and that for the *Rgs10-/-* is 200 bp.

### Mouse Infection Model

Equal number of female and male WT and *Rgs10-/-* mice, which were studied at eight- to ten-weeks of age, were anesthetized by intraperitoneal injection of Avertin (2,2,2-tribromoethanol (TBE)) (250 mg/kg). A negative response to a toe pinch confirmed adequate anesthetic depth. Mice were then inoculated intranasally (i.n.) with 100 plaque-forming unit (PFU) of mouse-adapted PR8 (A/Puerto Rico/8/1934 (H1N1)) influenza A virus in 50 µl of 1X-phosphate buffered saline (PBS). The infected mice were monitored daily for weight loss. In accordance with institutional guideline at the time of conducting the experiments, mice that lost >20% of their body weight were considered moribund and thus euthanized. All the animal experiments and the procedures were approved by Institutional Animal Care and Use Committee at The University of Georgia (protocol ID: A2020 11-001-Y1-A3).

### Cell Lines

The Madin-Darby canine kidney (MDCK)-Atlanta cell line is a generous gift from Dr. Stephen Tompkins lab at University of Georgia (Athens, GA). MDCK-Atlanta cells were grown in HyClone Dulbecco’s Modified Eagle Medium (DMEM)/F12 1:1: Liquid (Cytiva, cat#: SH30126.01) supplemented with 10% BenchMark™ heat-inactivated fetal bovine serum (Gemini Bio, cat#: 100-106), 1% of L-Glutamine Solution (Gemini Bio, cat#: 400-106), and an antibiotics combination of (1% penicillin/streptomycin) (Gemini Bio, cat#: 400-109) and incubated at 37°C in a humidified atmosphere 5% CO_2_.

### Influenza Virus Propagation and Purification

The A/Puerto Rico/8/1934(H1N1) influenza virus strain (referred to as PR8) was grown in the allantoic cavity of 9- to 10-day old specific pathogen-free embryonated chicken eggs at 37°C. The allantoic fluids were collected and then cleared by low-speed centrifugation. The viral supernatants were subsequently overlaid onto a 25% sucrose cushion in NTE buffer (100 mM NaCl, 10 mM Tris, 1 mM EDTA) and pelleted by ultracentrifugation in a Beckman Coulter SW32 rotor at 28,000 rpm for 2 hours. The purified PR8 viruses were resuspended in 2 mL of 1XPBS buffer, aliquoted, and frozen at − 80 °C. The viral stock titer was determined by plaque assay on monolayers of MDCK-Atlanta cells and hemagglutination assay, as described previously (25).

### Bronchoalveolar Lavage Fluid (BALF) Collection and Total Protein Analysis

The mice were first anesthetized and euthanized. After the trachea was exposed, the airways of euthanized mice were washed three times with 1 ml of sterile PBS, and the collected BALF was centrifuged at 400 x g for 10 min at 4°C. Soluble protein analytes of mouse BALF from WT and *Rgs10 -/-* mice were collected and subsequently preserved with addition of 1 mM PMSF and 1X proteases/phosphatase inhibitor cocktail (Cell Signaling technology, cat#: 5872S) before storage at − 80 °C for protein, chemokine, and cytokine detection. The concentration of proteins was measured using Pierce™ BCA Protein Assay Kit (Thermo Fisher Scientific, cat#: 23225).

### Immune Cell Phenotyping by Flow Cytometry

After lysing red blood cells using ammonium-chloride-potassium (ACK) lysis buffer (Thermo Fisher Scientific, cat#: A1049201) and two 1X PBS wash step, leukocytes in the BALF pellet were resuspended in 1 ml sterile 1X PBS and counted. For phenotyping, 1X10^6^ cells were suspended in 100 µl of 1X PBS and stained with Zombie Aqua™ flexible viability dye (1:100, BioLegend, Cat#: 423101) at room temperature in the dark for 15 mins. Following one washing step, the cells were suspended in 100 µl of 1X PBS containing (1% bovine serum albumin) and blocked using TruStain FcX™ anti-mouse (CD16/32) antibody (BioLegend, Cat#: 101320) on ice and protected from the light for 10 mins. The cells were subsequently stained with the following surface antibodies: ‘myeloid antibody cocktail’ [CD11b-PE/Cy7 (Cat#: 101216), CD115-APC (Cat#: 135510), CD11c-BV605 (Cat#: 117334), Ly6G-AF488 (Cat#: 127626), Ly6C-APC/F750 (Cat#: 128046), and F4/80-PE (Cat#: 123110)] and ‘lymphoid antibody cocktail’ [CD45-APC/F750 (Cat#: 103154), CD3-APC (Cat#: 100236), CD4-AF488 (Cat#: 100423), CD8a-AF700 (Cat#: 100766), B220-BV785 (Cat#: 103246), and NK 1.1-PE (Cat#: 108708)], all from Biolegend (San Diego, CA) at a 1:100 dilution at the same time to detect the number of cells from each of the myeloid and lymphoid cell subsets. Samples were read within 12 hours using a NovoCyte Flow Cytometer (ACEA Biosciences, Inc.) with the NovoExpress^®^ software at the University of Georgia College of Veterinary Medicine Cytometry Core Facility. Data acquisition was followed by analysis using FlowJo v.10.6.1 (BD Biosciences, San Jose, CA).

### Cytokine and Chemokine Analysis

The magnetic bead-based Bio-Plex assay was used to measure levels of 31 different mouse cytokines and chemokines in the BALF (stored at -80°C as indicated above) using internal standards (Bio-Plex Pro Mouse Chemokine Panel 31-plex, Bio-Rad, Cat#:12009159) following manufacturer’s instructions. The assay was performed at the University of Georgia CTEGD Shared Resources Laboratory on a Bio-Plex 3D analyzer (Bio-Rad Inc., Hercules, CA). Interferon-alpha enzyme-linked immunosorbent assay kit (Invitrogen, Cat#: BMS6027) was used to measure INFα levels in the BALF following the manufacturer’s protocol.

### Lung Viral Titration

Lung tissues from infected WT and *Rgs10 -/-* mice were collected and homogenized in 1 ml 1X PBS using GentleMacs Dissociator (Miltenyi Biotech, Cat#: 130-093-235). Lung homogenates were centrifuged at 5,000 x g for 15 mins at 4°C, and virus-containing supernatants were collected and stored at -80°C. Lung viral titers were determined by plaque assay on MDCK-Atlanta cells that were infected by serial, 10-fold dilutions of infected lung homogenate supernatants in DMEM/F12 (1:1) medium-containing 1 µg of trypsin, TPCK treated (Worthington Biochemical, Cat#: LS003740). Following 1 hour of MDCK-Atlanta cells infection, prepared overlay medium containing 1% Avicel in a ratio 1:1 was applied to infected monolayers of MDCK-Atlanta cells that were subsequently cultured at 37°C in a humidified atmosphere 5% CO_2_ for 72 hours. Cells were then washed with 1X PBS three times and stained with crystal violet, and viral plaques were counted. Viral titers were calculated and presented as lung viral titer (PFU, Log_10_).

### Histopathology Evaluation

WT and *Rgs10-/-* mice were sacrificed as indicated above, and murine lungs were intratracheally inflated with 1 ml of 10% Neutral Buffered Formalin. The trachea was tightened, and lungs were collected into 10% Neutral Buffered Formalin. Formalin-fixed/paraffin-embedded lung tissue blocks were prepared by the Histology Laboratory at the College of Veterinary Medicine at UGA and were sectioned at 5 µm thickness. Lung tissue sections were either left unstained for subsequent immunofluorescence experiments or stained with hematoxylin and eosin (H&E). H&E slides were analyzed by a pathologist who was blinded to the experiments. The lung histological score was determined on a scale from 0 to 4 based on a combination of assessments including bronchioles and alveolar space structure and infiltration of inflammatory cells and aggregation in alveoli, bronchioles, interstitium, blood vessels and pleura. A score of 0 represented no damage; 1 represented mild damage; 2 represented moderate damage, 3 represented severe damage; and 4 represented very severe damage and marked histological changes as done previously (25).

### Immunofluorescence

Lung tissue sections were deparaffinized, and rehydrated with xylene and alcohol gradient. Sections were antigen-retrieved with 0.1 M sodium citrate in Pascal Pressure Cooker (DakoCytomation, Aligent Technologies, Santa Clara, CA) for 20 min at 95 °C and then permeabilized with 0.1% Triton X100 while blocking in PBS with 5% BSA and 10% normal horse serum for 1 h. Primary antibody staining to detect Influenza A Virus Nucleoprotein (NP) (BEI Resources, Cat# NR-43899) was performed at a 1:250 dilution in PBS with 1% BSA, 1% normal horse serum, and 0.3% Triton X100 overnight at 4 °C. Sections were washed in 1X-TPBT, and secondary antibody staining was performed at a 1:1,000 dilution in PBS with 1% BSA, 1% normal horse serum for 1 h with horse anti-mouse IgG antibody (H+L), fluorescein (Vector Laboratories, cat#: FI-2000-1.5). Sections were washed with 1X-TPBS and then vectashield™ anti-fade mounting medium with DAPI (Vector Laboratories, cat#: H-1200-10) was applied to the lung tissue sections prior coverslip addition. All digital images were acquired at the University of Georgia College of Veterinary Medicine Cytometry Core on a Nikon A1R confocal microscope (Nikon Eclipse Ti-E inverted microscope) and examined with NIS Element software (Nikon, Version 6.4).

### Quantitative Real-Time Polymerase Chain Reaction

Total RNA was isolated from lung homogenate using TRIzol reagent (Invitrogen, Cat#: 15596018). cDNA was synthesized from 2 μg of total RNA using the High-capacity Reverse Transcriptase cDNA kit (Applied Biosystem, Cat#: 4368814). Each cDNA sample was diluted 10-fold, and 5 µl was used in a 15 µl PCR reaction (SYBR™ Green PCR Master Mix.) (Thermo Fisher Scientific, cat#: 4309155) containing primers at concentration of 5 µM each. All the reactions were run in triplicates. The mRNA expression levels were normalized to the housekeeping β-actin gene and were calculated using the *2−* ^ΔΔCT^ method. Mouse *Rgs2*, *Rgs10*, *Rgs12*, *Rgs14* and β-actin primers were obtained from Millipore Sigma. The primer sequences used for gene amplification are listed as follows: *Rgs2* forward, 5′- GAGAAAATGAAGCGGACACTCT-3’, *Rgs2* reverse, 5′- GCAGCCAGCCCATATTTACTG-3’, *Rgs10* forward, 5’-CCCGGAGAATCTTCTGGAAGACC-3’, *Rgs10* reverse, 5’-CTGCTTCCTGTCCTCCGTTTTC-3’, *Rgs12* forward, 5′- GTGACCGTTGATGCTTTCG-3’, *Rgs12* reverse, 5′- ATCGCATGTCCCACTATTCC-3’, *Rgs14* forward, 5′- AAATCCCCGCTGTACCAAG-3’, *Rgs14* reverse, 5′- GTGACTTCCCAGGCTTCAG-3’, *Actb* forward, 5′- GGCTGTATTCCCCTCCATCG-3’, *Actb* reverse, 5′-CCAGTTGGTAACAATGCCATGT-3’.

### MPO-DNA ELISA

MPO-DNA complexes were detected in the BALF of infected WT and *Rgs10-/-* mice following a published protocol (26, 27) adapted to murine samples (28). BALFs were diluted 1:25 in PBS and were subsequently added to a high-binding 96-well ELISA microplate pretreated overnight at 4 °C with capture anti-MPO (1:200 RD Systems, Cat#: AF3667). The wells of microplate were blocked with 5% BSA in PBS at room temperature for 2 hours. Samples were incubated overnight at 4 °C. Following three washes with PBS/Tween-20, the secondary mouse anti-DNA-POD (HRP-conjugated anti-DNA antibody, 1:500, Roche, Cat#: 1154467501) was added for 1 h at room temperature. Samples were washed four times with PBS/Tween 20. TMB substrate (Thermo Scientific, Cat#34021) was added, and the reaction was stopped by the addition of 1 M HCl. Absorbance was measured at 450 nm with an Eon microplate spectrophotometer (BioTek, Winooski, VT). Background absorbance values of the medium were subtracted. All samples being compared were run in duplicates on the same plate. Differences between optical densities were compared.

### Neutrophil Elastase Activity

Neutrophil elastase (NE) enzymatic activity of the IAV-infected BALFs was determined using the NE activity assay kit that uses a specific NE substrate (Z-Ala-Ala-Ala-Ala) 2Rh110 (Cayman Chemical, cat#: 600610). The NE enzymatic activity in BALFs was determined by measuring NE-dependent cleavage of 2Rh110 to highly fluorescent compound R110. This fluorescence intensity was read using an excitation of 485 nm and emission wavelength of 525 nm. The standard curve was generated using human NE at 18 U/ml and were serial diluted from 10 mU/ml - 0 mU/ml in assay buffer provided with the kit. The substrate was added to each well and put in the Varioskan Flash™ microplate fluorimeter (Thermo Fisher Scientific, Waltham, MA). The plate was then incubated at 37°C throughout the fluorescence readings and read for 1.5 hours. Fluorescence measurements were taken every 2 minutes for 1.5 hours, and the average fluorescence for each well was measured. To calculate the NE concentration in each sample in mU/ml, the average fluorescence for each standard was calculated, and the blank was subtracted from each well. Based on the standard curve generated: NE (mU/ml) = [(fluorescence – (y-intercept))/slope] x dilution factor.

### Statistical Analysis

All quantified data were analyzed for statistical difference between groups using unpaired student t-test (for comparing two groups) or one-way ANOVA followed by Tukey *post-hoc* analysis (for comparing three or more groups). GraphPad Prism software was used to carry out statistical analyses. Data are expressed as mean ± S.E.M. where *, p<0.05; **, p<0.01; and ***, p<0.001 indicate the levels of significance. The relevant statistical information including *n* values and p-values are given in individual figure legends.

## Results

### *Rgs10-/-* Mice Exhibit Enhanced Mortality and Weight Loss Post-IAV Infection

**Figure 1:**
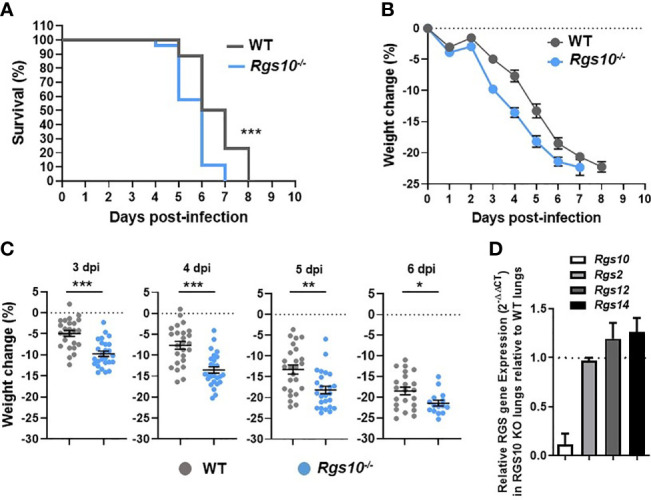
*Rgs10-/-* mice exhibit enhanced mortality and weight loss post-IAV infection. WT (n=25) and *Rgs10-/-* (n=26) mice were inoculated intranasally with 100 PFU of the influenza A/Puerto Rico/8/1934 H1N1 (PR8) virus and **(A)** survival and **(B)** average weight changes were monitored for up to 8 days. **(C)** Individual animal weight changes are shown for each day between 3 to 6 dpi. Each circle represents a separate animal. The data were pooled from two independent experiments. Data were analyzed for statistical differences using Mann–Whitney U test. Data are presented as mean ± SEM where *p < 0.05; **p < 0.01; ***p < 0.001. **(D)** RNA was isolated from the lung homogenates of uninfected wild-type (WT) and *Rgs10-/-* mice (n=3 per strain), *Rgs10, Rgs2, Rgs12* and *Rgs14* transcript levels were measured using quantitative RT-PCR and normalized on the endogenous housekeeping gene β-actin. The relative expression was calculated by using the 2-ΔΔCt method and the gene expression ratio of *Rgs10-/-* over WT mice are shown for each gene. The dashed line indicates endogenous expression in uninfected WT lungs.

While previous studies have investigated the impact of RGS10 on the pathophysiology of diverse disease models using *Rgs10-/-* mice (13), no work has studied the *in vivo* role of RGS10 in fighting infections. To explore the *in vivo* role of RGS10 in response to viral challenge, eight-to-ten-week-old, sex- and weight-matched *Rgs10-/-* mice and their *Rgs10*-expressing C57BL/6 wild-type (WT) littermate control mice were infected intranasally (i.n.) with a lethal dose of 100 plaque-forming units (PFU) of the mouse-adapted influenza A/Puerto Rico/8/1934 H1N1 (PR8) virus. While most people infected with influenza do not die [its mortality rate is around 0.1% (1)], we decided to study the role of RGS10 in a murine model of lethal influenza infection because severe infections are associated with worse clinical outcomes requiring hospitalization and expensive treatments, mainly in immunocompromised patients. Survival and body weight loss were monitored for eight days. *Rgs10-/-* mice demonstrated significantly increased mortality compared to WT animals (**Figure 1A**). Both WT and *Rgs10-/-* mice started losing body weight gradually from day 3 post-infection (**Figure 1B**), but *Rgs10-/-* mice lost significantly more body weight compared to WT mice (3 through 6-day post-infection, dpi) (**Figure 1C**). In addition to prior published data using western blotting (13), the absence of Rgs10 expression was also confirmed in this study using qRT-PCR in lungs extracted from *Rgs10-/-* mice (**Figure 1D**). No effect of *Rgs10* deficiency was observed on the transcript expression of *Rgs2*, the widely expressed RGS protein with high expression in airways, or on the transcript levels of *Rgs12* and *Rgs14*, which share high sequence identity with the RGS domain of RGS10 protein (**Figure 1D**). These results indicate that RGS10 delays the onset of the clinical consequences of lethal respiratory IAV infection.

### *Rgs10* Deficiency Promotes Severe Lung Damage During IAV Airway Infection

**Figure 2:**
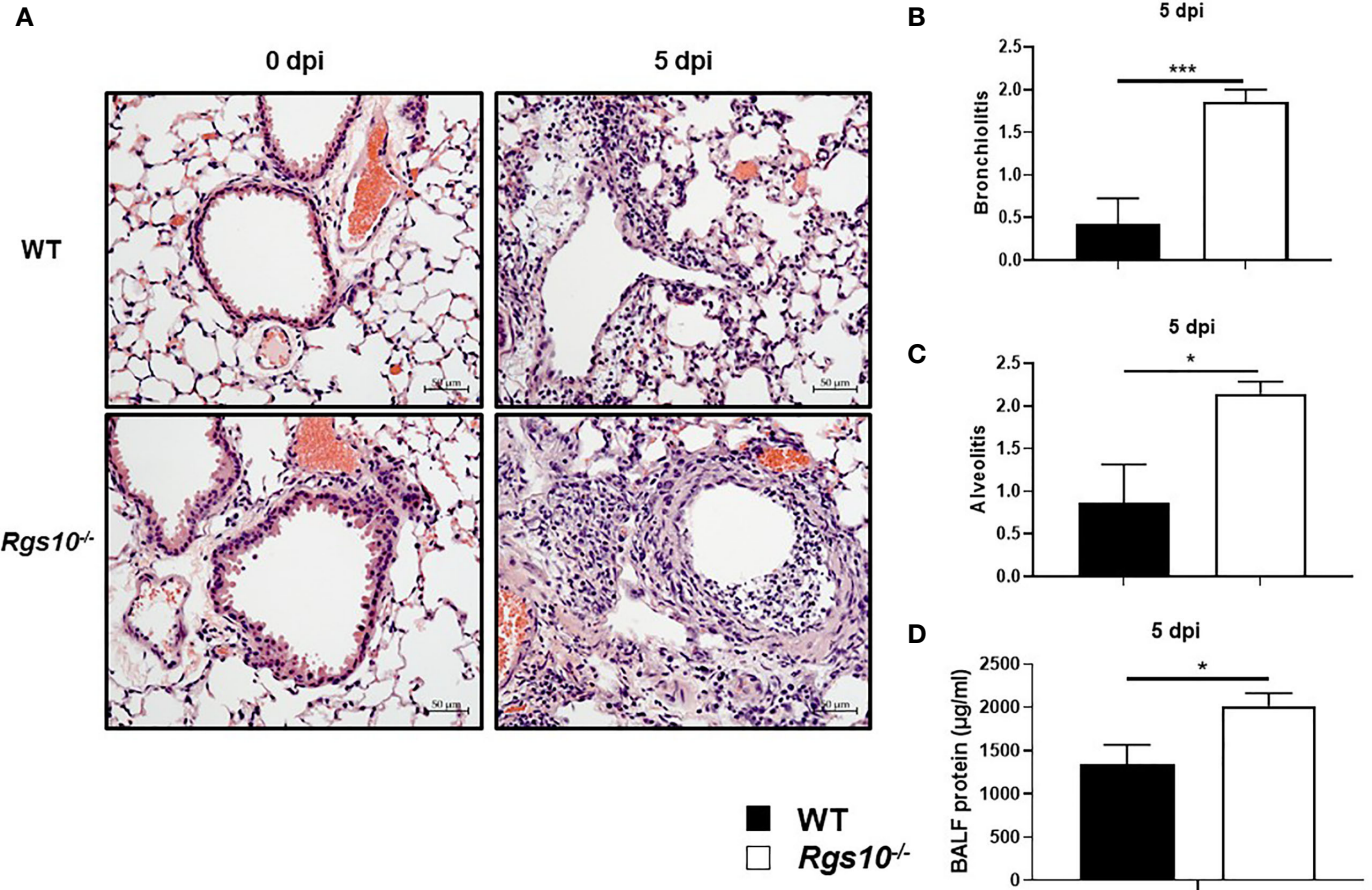
*Rgs10* deficiency promotes severe lung damage during IAV airway infection. Lung histopathological changes and BALF protein concentrations were evaluated in uninfected (0 dpi) WT and *Rgs10-/-* mice (n=5) or WT and *Rgs10-/-* mice (n=7) infected intranasally with 100 PFU of PR8 virus at 5 dpi. **(A)** Paraffin-embedded lung sections from WT and *Rgs10-/-* mice at 0 (uninfected) and 5 dpi were stained with hematoxylin and eosin (scale bar=50 μm). Representative images of at least five similar results are shown. Lung histopathological scores were assessed in or around bronchioles (bronchiolitis) **(B)** or alveolar spaces (alveolitis) **(C)**. **(D)** Total protein concentrations in BALFs of WT and *Rgs10-/-* mice were determined at 5 dpi. Data were analyzed for statistical differences using unpaired t-test between groups. Data are presented as mean ± SEM where *p < 0.05; ***p < 0.001.

Next, we investigated whether the increased weight loss and death resulting from IAV infection in *Rgs10-/-* mice were associated with more severe lung damage. To test this, we inoculated WT and *Rgs10-/-* mice i.n. with 100 PFU influenza virus, and 3 and 5 dpi, we assessed the pathological changes in the lung tissues. We chose these time points (3 and 5 dpi) to assess lung injury because 3 dpi showed the earliest difference in weight loss between genotypes, and 5 dpi showed the earliest and largest difference in survival between strains. Approximately 42.3% of *Rgs10-/-* mice died at 5 dpi (survival rate, 57.7%) in response to 100 PFU PR8 infection compared to 11.5% of mortality rate in infected WT mice. Following IACUC guidelines and the approved protocol at UGA, mice that lost greater than 20% of their initial weight at day of infection were considered moribund and subsequently removed from the ongoing experiment. To examine the lung histopathology of uninfected animals or infected mice at 3 and 5 dpi, lung sections collected from WT and *Rgs10-/-* mice stained with hematoxylin and eosin (H&E). No histopathological differences were observed in the lungs between uninfected (0 dpi) WT and *Rgs10-/-* mice (**Figure 2A**). Despite the significant weight loss in infected *Rgs10-/-* mice, no differences in the signs of lung damage were observed between WT and *Rgs10-/-* mice at 3 dpi (**Supplementary Figures S1A, B**). More importantly, at 5 dpi, *Rgs10-/-* mice developed severe lung damage demonstrated by massive inflammatory cell infiltration, such as neutrophils, into the bronchioles (bronchiolitis) (**Figures 2A, B**) and inflammatory cells aggregation into the alveolar walls and spaces (alveolitis) (**Figures 2A, C**). Unlike *Rgs10-/-* mice, infected WT mice only showed minimal bronchial cell infiltrates and alveolar cell aggregates (**Figures 2A**–**C**). To further assess lung damage, we measured the total protein concentration in BALFs, as an indicator of general inflammation and epithelial leakage. There was no significant difference in the BALF protein levels between WT and *Rgs10-/-* mice at 0 dpi (**Supplementary Figure S1C**). However, the results showed that protein concentrations were significantly higher in the BALFs of PR8-infected *Rgs10-/-* mice at 3 dpi (1,152 ± 280.2 μg/ml) (**Supplementary Figure S1C**) and at 5 dpi (2,019 ± 144.1 μg/ml) (**Figure 2D**) compared to their WT control counterparts (826.9 ± 154.2 μg/ml) (**Supplementary Figure S1C**) and (1,339 ± 228.6 μg/ml) (**Figure 2D**), respectively. Altogether, the absence of Rgs10 is associated with increased tissue damage, bronchiolitis, alveolitis and general inflammation in influenza-infected mice.

### *Rgs10* Deficiency Increases Viral Titers in IAV-Infected Lungs

**Figure 3:**
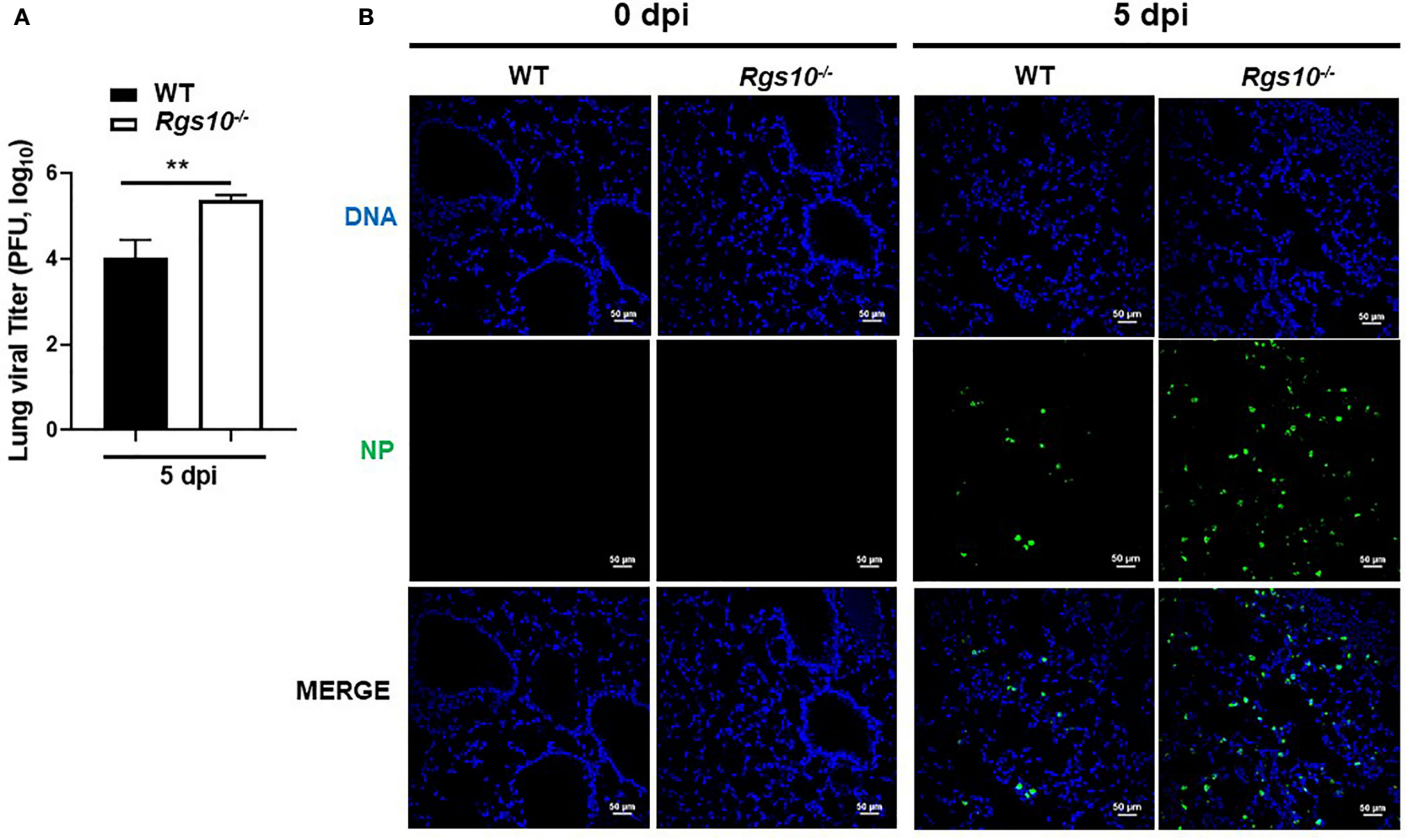
*Rgs10* deficiency increases viral titers in IAV-infected lungs. **(A)** WT and *Rgs10-/-* mice (n=6) were inoculated intranasally with 100 PFU of PR8 virus. Lungs were harvested from infected strains, and homogenates were prepared for viral titer assay. Comparison of lung viral loads between stains on 5 dpi were determined using plaque assay. **(B)** WT and *Rgs10-/-* mice (n=6) were infected intranasally with 100 PFU of PR8 virus as in **(A)**. Lungs were fixed, and immunofluorescence staining was performed on infected lung sections using an anti-nucleoprotein (NP) antibody identifying influenza A virus NP (green) and DAPI (blue) to detect DNA. Representative images (scale bar=50 μm) of at least five similar results are shown. Data were analyzed for statistical differences using unpaired t-test between groups. Data are presented as mean ± SEM **p < 0.01.

To determine if the increased inflammatory response-mediated lung injury of *Rgs10-/-* mice correlated with higher viral loads, we measured lung virus titers using the plaque assay. WT and *Rgs10-/-* mice were infected as described above. At 5 dpi, infected lungs were collected and processed to determine the lung viral titers using MDCK-Atlanta cells. Viral loads in *Rgs10-/-* mouse lungs were significantly higher (1.4 log difference) than the viral loads in WT mouse lungs (*P*=0.0097) (**Figure 3A**). To further confirm that *Rgs10-/-* mouse lungs retain higher viral loads following IAV infection, sectioned lungs from PR8-infected WT and *Rgs10-/-* mice at 5 dpi were probed with anti-IAV nucleoprotein (NP) antibody. The results confirmed increased viral loads in the lungs of *Rgs10-/-* mice compared to WT littermate controls (**Figure 3B**). Overall, these findings indicate that lack of RGS10 results in elevated viral titers in the lungs following IAV infection.

### *Rgs10* Deficiency Elevates Cytokine and Chemokine Levels Upon IAV Infection

**Figure 4:**
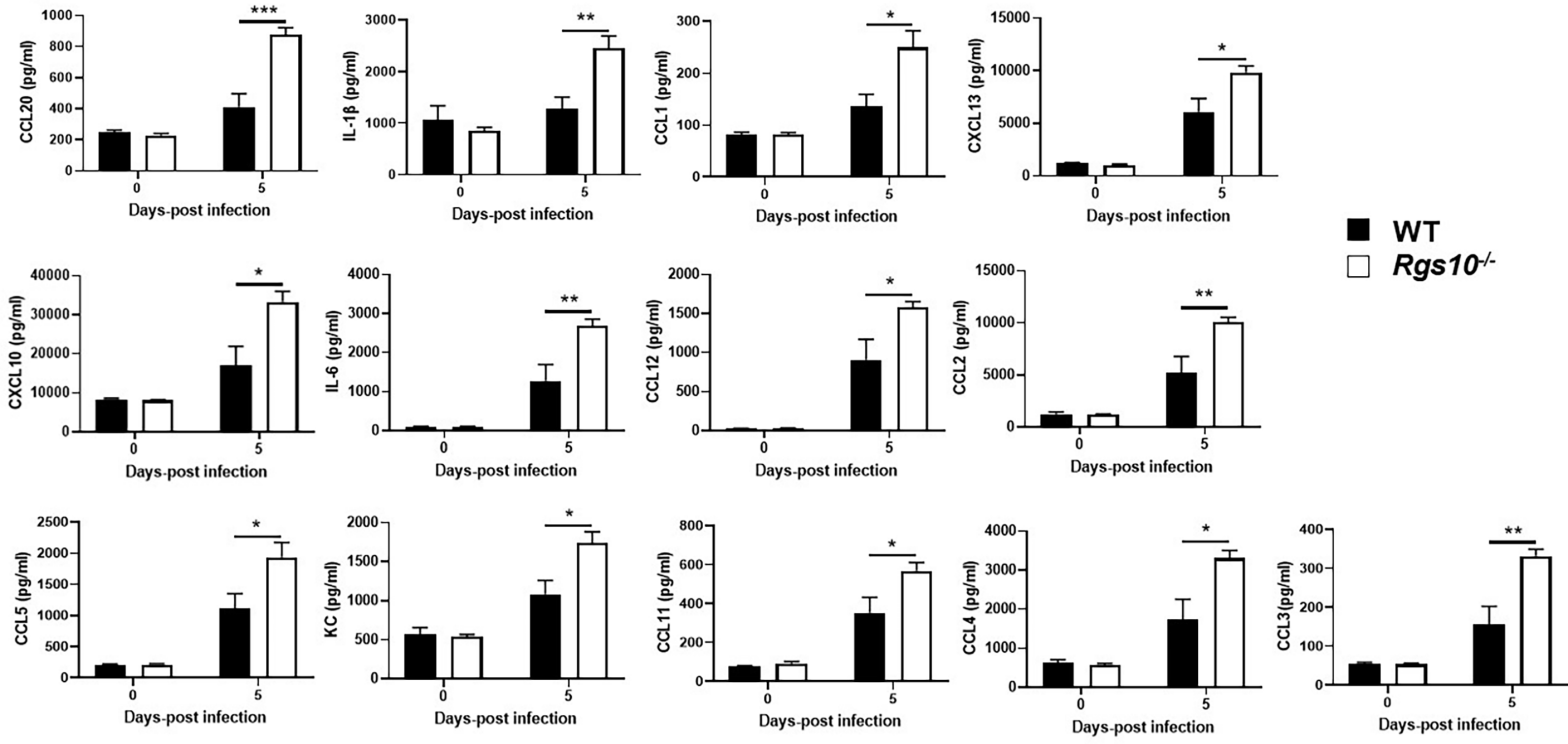
*Rgs10* deficiency elevates cytokine and chemokine levels upon IAV infection. The bead-based Bio-Plex assay was used to measure cytokine and chemokine levels in the BALF supernatants of uninfected (0 dpi) WT and *Rgs10-/-* mice (n=5) or WT and *Rgs10-/-* mice (n = 7) infected intranasally with 100 PFU of PR8 virus at 5 dpi. Data were analyzed for statistical differences using an analysis of variance (ANOVA) followed by Tukey *post hoc* test between groups. Data are presented as mean ± SEM where *p < 0.05; **p < 0.01; ***p < 0.001.

Dysregulation of the inflammatory response is a major factor by which IAV infection causes morbidity and mortality (29). Because of the increased protein concentrations and viral loads in *Rgs10-/-* airways, we next quantified cytokine and chemokine levels in BALF collected from uninfected (0 dpi) or PR8-infected WT and *Rgs10-/-* mice. In general, there were no observed differences in the BALF levels of any cytokines and chemokines between uninfected (0 dpi) WT and *Rgs10-/-* mice (**Figure 4** and **Supplementary Figure S2**). At 5 dpi, *Rgs10-/-* mice demonstrated considerably higher levels of certain cytokines and chemokines including IL-1β, IL-6, CCL1, MCP-1 (CCL2), CCL3, CCL4, CCL5, CCL11, CCL12, CCL20, KC (CXCL1), CXCL10, and CXCL13, respectively (**Figure 4**). There were elevated, but not significantly different levels of GM-CSF, IFN-γ, CCL7, CCL19, CCL22, and CCL24 (**Supplementary Figure S2**), and no differences were observed in several other cytokine and chemokine levels (**Supplementary Figure S2**). Thus, these data suggest that deficiency of RGS10 is implicated in shaping the immune response by enhancing the release of inflammatory cytokines and chemokines in infected mouse lungs.

### *Rgs10* Deficiency Increases Neutrophil and Monocyte Recruitment Into the Airway During IAV Infection

**Figure 5:**
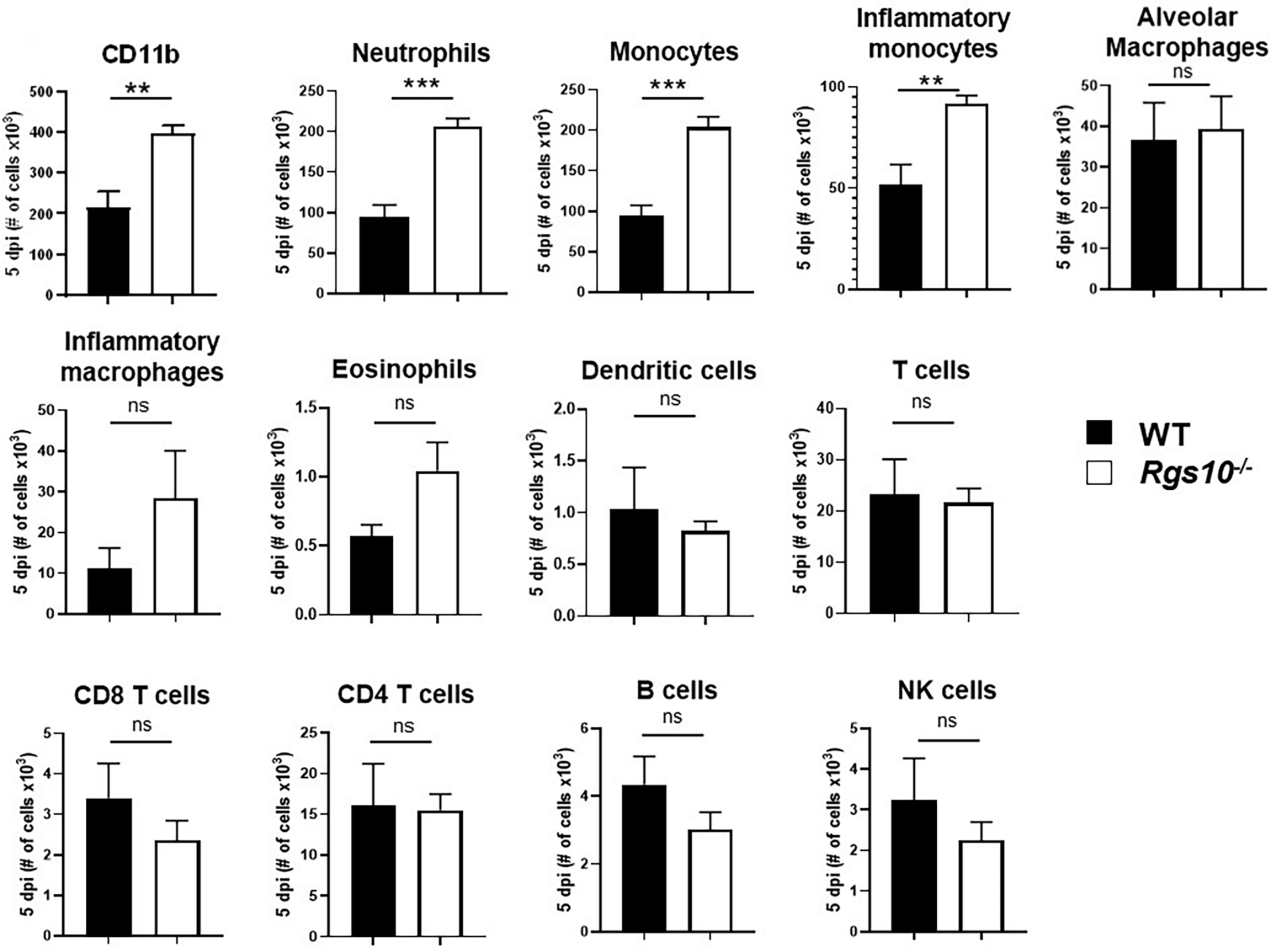
*Rgs10* deficiency increases neutrophil and monocyte recruitment into the airway during IAV infection. Phenotypic analysis of the indicated myeloid and lymphoid leukocyte subsets was measured by flow cytometry in PR8-infected lung BALF of WT and *Rgs10-/-* mice (n = 7) at 5 dpi. Data were analyzed for statistical differences using unpaired t-test between groups. Data are presented as mean ± SEM where **p < 0.01; ***p < 0.001, n.s. not significant.

To determine whether the increased levels of cytokines and chemokines manifested in increased numbers of particular leukocyte subsets, we characterized the composition of infiltrating and residential immune cells in the BALF of both uninfected or PR8-infected WT and *Rgs10-/-* mice at 3 and 5 dpi. Flow cytometry was performed using surface markers to distinguish and determine the numbers of myeloid (neutrophils, monocytes, inflammatory monocytes, eosinophils, dendritic cells, alveolar (pulmonary) macrophages and inflammatory (monocyte-derived) macrophages) (**Supplementary Figure S3**) and lymphoid (T cells, B cells, and natural killer (NK) cells) (**Supplementary Figure S4**) cell subsets. As expected, alveolar macrophages represented the dominant immune cell type in uninfected airways (**Supplementary Figure S5A**). There were no differences in the number of CD11b^+^ leukocytes or in the number of any subset of myeloid and lymphoid cells between mouse strains without infection (**Supplementary Figure S5**) or at 3 dpi (**Supplementary Figure S6**). However, at 5 dpi, the total number of CD11b^+^ cells was about two times higher in *Rgs10-/-* mice compared to WT animals (**Figure 5**). This significant increase in total CD11b^+^ leukocyte counts was most likely accounted for by significantly higher numbers of neutrophils, monocytes and inflammatory monocytes in PR8-infected *Rgs10-/-* mice in comparison to infected WT animals (**Figure 5**). Analysis of BALF from WT and *Rgs10-/-* mice at 5 dpi exhibited no statistically significant differences in the number of eosinophils, dendritic cells, alveolar macrophages, inflammatory macrophages, T cells, B cells and NK cells (**Figure 5**). Collectively, the increased susceptibility to IAV challenge in *Rgs10-/-* mice is associated with excessive influx of neutrophils and monocytes to the lungs.

### Neutrophil Activation Markers Are Higher in the Lungs of PR8-Infected *Rgs10-/-* Mice

**Figure 6:**
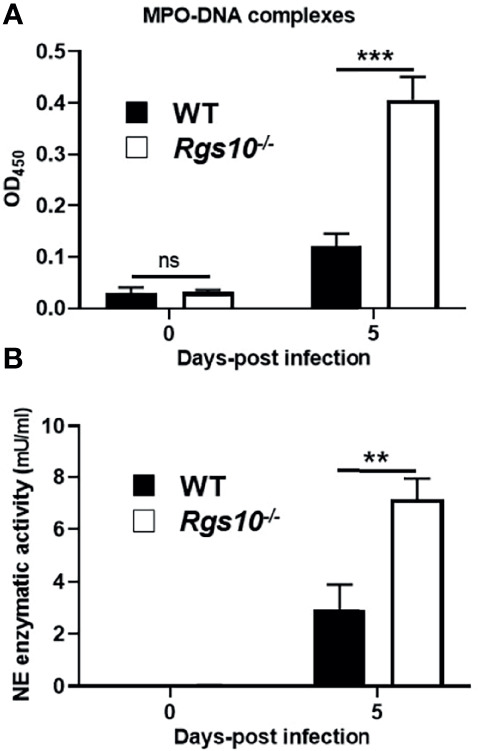
Neutrophil activation markers are higher in the lungs of PR8-infected *Rgs10-/-* mice. Neutrophil extracellular traps were measured in collected lung BALFs from uninfected (0 dpi) and infected WT and *Rgs10-/-* mice (n = 7) with 100 PFU of PR8 virus **(A)** at 5 dpi using ELISA that detects MPO-DNA complexes, indicative of NETs. **(B)** Neutrophil elastase enzymatic activity was detected in cell-free BAL supernatants of mice at 0 and 5 dpi by using the NE-specific fluorogenic substrate. Data were analyzed for statistical differences using unpaired t-test between groups. Data are presented as mean ± SEM where **p < 0.01; ***p < 0.001.

Neutrophils have previously been shown to have a protective role following influenza virus infection (30). However, growing evidence strongly implicates that accumulation of neutrophils following severe infections can excessively release NETs, which trigger inflammatory responses and result in tissue injury-mediated organ dysfunction (31). Because of the abundant neutrophil infiltrates in infected airways of *Rgs10-/-* mice, we explored whether soluble markers of NETs are also increased in the lungs of *PR8*-infected *Rgs10-/-* mice. Cell-free BALF supernatants were collected from WT and *Rgs10-/-* mice at 0 and 5 dpi and the presence of NET-specific MPO-DNA complexes was assessed as previously (26–28). While there was no significant difference in the BALF MPO-DNA complexes level between WT and *Rgs10-/-* mice at 0 dpi, we found that there was a significant, 4-fold increase in the absorbance values in the BALFs of infected *Rgs10-/-* mice compared to their WT littermate controls (**Figure 6A**), indicating the enhanced release of NETs in infected airways of these animals. To further characterize the consequences of neutrophils’ presence in infected *Rgs10-/-* lungs, we measured NE enzymatic activity, one of the key enzymes in neutrophils and NETs (32). Our data revealed that PR8-infected *Rgs10-/-* lungs have significantly higher BALF NE activity (7.2 mU/ml) than their WT control groups (2.9 mU/ml) (**Figure 6B**). Therefore, the above results demonstrate more NETs and NE released in the airways of *Rgs10-/-* mice that likely contribute to the observed tissue damage in the lung.

## Discussion

RGS10, highly expressed in immune cells, has a critical role in the regulation of immune cell activation and inflammatory responses. More specifically, RGS10 suppresses NF-*κ*B activation and the expression of inflammatory genes such as TNF-α, interleukins, COX-2 and iNOS in multiple types of (resident and recruited) macrophages (18–20, 23, 24). Several studies have investigated the role of RGS10 in inflammation-associated diseases, such Parkinson’s disease (23), multiple sclerosis (33), thioglycolate-induced peritonitis (19), osteopetrosis (34–36), cardiac hypertrophy (37), chemoresistant ovarian cancer (38–41), platelets aggregation and thrombogenesis (42–46), obesity and related metabolic syndromes (47), rheumatoid arthritis (48), and colitis-related neurologic dysfunction (49).

Even though RGS10 has been extensively studied in multiple animal and cellular models of these diseases, the biological function of RGS10 has not yet been explored in any infectious disease including respiratory viral infections. Our study is the first to investigate the *in vivo* physiological role of RGS10 in a mouse model of respiratory IAV infection and in general, any infectious disease. Influenza infections are characterized by cytokine storm and overactive innate immune cells that cause lung tissue damage and contribute to disease morbidity and mortality (50, 51). The findings presented in this study show that RGS10 provides improved protection to the host against lethal influenza-stimulated lung inflammation and pathology, as loss of functional RGS10 protein leads to a significantly worse scenario upon IAV infection. In this study we used a lethal influenza infection mouse model because severe influenza is associated with hospitalization, severe lung disease, death and represents a significant economic burden (1). Therefore, it is the most urgent to understand the disease pathogenesis in this specific, more susceptible patient population and to identify new therapeutic targets with the potential largest clinical impact. Future studies will, however, include investigations on the role of RGS10 in nonlethal influenza infections to better understand its role under different infectious conditions.

*Rgs10-/-* mice showed increased susceptibility to lethal infection with the PR8 H1N1 virus exhibiting increased weight loss and mortality associated with delayed viral clearance as well as exacerbated lung inflammation and tissue damage. Lung infection with IAV is established to cause dysregulation of innate immune cells that leads to extensive release of cytokines and chemokines that subsequently contribute to severe lung damage (51, 52). In the context of PR8 challenge, the absence of Rgs10 resulted in elevated levels of several proinflammatory cytokines and chemokines in the BALF. The most significant difference in BALF levels between *Rgs10-/-* and WT mice was observed in case of CCL20. CCL20 is mainly released by airway epithelial cells but also by activated monocytes and neutrophils and mediates the migration of CCR6^+^ lymphoid and dendritic cells (53, 54). While BALF levels of dendritic cells, T or B cells were not significantly influenced by *Rgs10* deficiency upon influenza infection, CCL20 could have specifically attracted or stimulated Th17 cells known to be activated during influenza infection and to recruit neutrophils to the airways (55–57). Previous reports have shown that deficiency of RGS10 in activated microglia (brain-resident macrophages) and macrophages including bone marrow-derived macrophages (BMDMs) and alveolar macrophages enhanced the transcript and protein levels of IL-1β and IL-6 (18–20). Loss of RGS10 in activated macrophages increased NLRP3 and NLRC4 inflammasome activity that could increase IL-1β release (58). Despite their reported antiviral effect, IL-1β and IL-6 have been implicated in pulmonary inflammation during influenza infection (59–61). CCL2 is known to be released by inflammatory monocytes and is chemotactic for a variety of immune cells, mainly monocytes (62). CCL3 also recruits different immune cells including monocytes and γδT cells (63, 64). CCL1, CCL4, CCL5, CCL11 and CCL12 are all members of the C-C chemokine family and known to recruit monocytes, but not neutrophils (65). CXCL1, CXCL10 and CXCL13 are members of the C-X-C chemokine family and are strongly chemoattractant towards neutrophils (66). All the aforementioned cytokines and chemokines with increased levels in *Rgs10-/-* BALFs are known to be induced during influenza infection in the airways (67). Overall, the documented, *Rgs10*-dependent increases in the BALF cytokine and chemokine levels are likely responsible for the enhanced recruitment of monocytes and neutrophils to influenza-infected airways and contribute to the increased mortality and morbidity. Based on this, we propose that RGS10 is a potentially important immune checkpoint of the early innate immune response to influenza that controls several leukocyte-recruiting and -activating molecules.

Flow cytometry analysis of the immune cells in the infected BALFs displayed a significant difference in the recruited cells in WT and *Rgs10-/-* mice. At day 5 post-PR8 infection, *Rgs10-/-* mice generated higher numbers of CD11b^+^ leukocytes relative to WT mice, reflecting the massive influx of the neutrophils and monocytes including inflammatory monocytes. The number of other immune cell subsets (lymphoid and myeloid) were comparable between the two mouse strains. This suggests that the enhanced viral titers observed in infected *Rgs10-/*- lungs seems to be independent of antiviral adaptive responses and dependent on innate immunity of neutrophils and monocytes.

Monocyte recruitment into the inflamed and IAV-infected lungs has been shown to induce more cytokines and chemokine that further augment lung inflammation and immune cell infiltration (68). In line with increased monocyte recruitment, the BALF levels of CCL2 (known as MCP-1) were higher in *Rgs10-/-* mice than in WT mice. CCL2 is the leading chemokine in monocytes and macrophages recruitment through the interaction with its receptor CCR2. Previous studies have implicated the pathological role of CCL2-CCR2 signaling in lung damage upon influenza virus infection (69). More specifically, the direct administration of the CCL2 blocking antibody resulted in decreased morbidity and mortality in response to IAV infection (70). Further, a CCR2 signaling antagonist reduced immunopathology and subsequently improved the survival following influenza virus infection in mice (71). Therefore, it is possible that one reason why *Rgs10-/-* mice do -in part- worse relative to WT animals is because of the hyperactivation of recruited monocytes, where RGS10 may have a potential role to control monocytes’ recruitment or activation.

Neutrophils, which are recruited rapidly -along with monocytes- to the lung following IAV infection, have been previously shown to have a protective role by enhancing host survival through their contribution to influenza virus clearance (72). However, massive recruitment of neutrophils to infected lungs by influenza virus can cause lung inflammation and pathology (67, 73–75). Partial neutrophil depletion leads to diminished pulmonary inflammation and decreased host morbidity (76). Blockade of CXCR2 signaling by anti-CXCR2 antibody administration has been shown to reduce neutrophil infiltration and thereby enhanced the survival of infected mice (77). CXCR2, which is functionally involved in neutrophil recruitment, is activated by the KC (CXCL1) chemokine (78–80). Consistent with increased numbers of infiltrated neutrophils, our results showed that PR8-infected *Rgs10-/-* lungs considerably secreted more KC (CXCL1) compared to infected WT lungs. This finding suggests that neutrophils are recruited to infected *Rgs10-/*- lungs -at least partially- *via* the CXCL1-CXCR2 axis. Data presented here also propose that RGS10 contributes to the prevention of exacerbated neutrophil airway recruitment during respiratory viral infections.

One of the neutrophils’ defense mechanisms against different microbes is the formation of NETs when activated neutrophils release fibers of extracellular DNA associated with histones and granule proteins like MPO and NE to entrap and kill extracellular pathogens (81, 82). However, sustained formation of NETs has been associated with severe tissue damage during influenza virus infection, and sustained activities of NE have been linked to tissue damage in inflammatory diseases of the lung (74, 83–88). Here, we report increased levels of NET-specific MPO-DNA complexes as well as the enzymatic activity of NE in PR8-infected *Rgs10-/-* BALFs. Thus, the pronounced lung damage in infected *Rgs10-/-* mice relative to infected WT mice could be due to the harmful effects of neutrophil-derived products.

Canonically, RGS10, as other RGS proteins, controls GPCR signaling by accelerating heterotrimeric G-protein inactivation *via* their intrinsic GTPase activity toward Gα-subunits. RGS10 selectively binds and terminates Gαi family G-proteins *via* its classic GAP activity (11, 14, 15). CCR2 and CXCR2 are Gαi protein-coupled receptors that facilitate monocyte and neutrophil recruitment and trafficking (89). Because of their classical roles in deactivation of G-protein signaling, a couple of studies have reported the regulation of neutrophil trafficking by RGS proteins. For example, the absence of Rgs2 in mice enhanced neutrophil infiltration into inflamed lungs (90, 91). Rgs2 is, however, unlikely responsible for the enhanced neutrophil infiltration seen here in *Rgs10-/-* animals as the *Rgs2* lung gene expression level remained unaffected by *Rgs10* deficiency. *Rgs5*-deficent mice also exhibited a massive influx of neutrophils in inflamed lungs (92). RGS10 is abundantly expressed in monocytes with low expression level in neutrophils (17, 93). It is highly likely that RGS10 controls IAV-triggered lung inflammation and pathology by limiting the canonical CXCL1-CXCR2 and CCL2-CCR2 axes, leading to reduced neutrophil and monocyte trafficking to the airways, respectively. While this work utilized a mouse model with global *Rgs10* deficiency, based on prior literature, we speculate that Rgs10 expression in pulmonary macrophages or monocytes must be significant contributors to the observed phenotype. Both cell types are known to express RGS10 that is down-regulated upon activation by microbial stimuli and to contribute to the anti-influenza immune response and related pathologies (13, 20). Increased proinflammatory cytokine release in pulmonary macrophages due to RGS10 deficiency could exacerbate the early innate immune response to influenza, even if pulmonary macrophage numbers remain Rgs10-independent (20). Further work is required to determine whether purified *Rgs10-/-* monocytes and neutrophils are hyperresponsive to chemokine or microbial signals.

RGS10 transcript and proteins levels are suppressed in several cells, including macrophages (19, 20), microglia (18, 23), neurons (94), cardiomyocytes (37), and ovarian cancer cells (38). Importantly, while RGS10 is highly enriched in macrophages and microglia under resting conditions, its expression is silenced in response to inflammatory stimuli, such as LPS and TNF-α. Loss of RGS10 in these cells contributes to multiple phenotypes, such as overactive microglia-mediated neuroinflammation and subsequent neurodegeneration (18, 23, 94), cardiac hypertrophy (37), and ovarian cancer chemoresistance (40), respectively. Our recent study identified the inflammatory responses that facilitate RGS10 suppression in activated macrophages and microglia, involving PI3K/NF-κB-dependent TNF-α and the activities of HDAC (1-3) enzymes (20). Given that RGS10 is suppressed in response to inflammatory signaling and the fact that IAV infection causes amplification of inflammatory mediators, such as cytokines, it will be interesting to investigate whether respiratory IAV infection suppresses RGS10 expression in airway epithelial cells or resident and recruited immune cells, and if so whether PI3K or NF-κB signaling pathways suppress its expression. Several cytokines with Rgs10-dependent BAL levels after influenza infection are also secreted by airway epithelial cells, not only by professional immune cells, further suggesting a potential role of epithelial Rgs10 in the process.

In summary, our results describe for the first time a phenotype for *Rgs10-/-* mice in terms of influenza virus infection. Deficiency of *Rgs10* in mice aggravates inflammatory response-induced lung pathology upon IAV infection, which consequently contributes to weight loss of infected mice. The significant difference in morbidity and mortality between *Rgs10-/-* and WT animals is likely due to elevated levels of cytokines and chemokines driving enhanced neutrophil and monocyte recruitments to the inflamed lung. The findings of this study propose RGS10 as a potential therapeutic target for respiratory IAV infections through limiting uncontrolled inflammation caused by overactive innate immune cells.

## Data Availability Statement

The original contributions presented in the study are included in the article/**Supplementary Material**. Further inquiries can be directed to the corresponding author.

## Ethics Statement

The animal study was reviewed and approved by Institutional Animal Care and Use Committee at The University of Georgia

## Author Contributions

FA and BR designed the experiments. FA, DS, ST, and KF conducted the experiments and performed data analysis. FA wrote the original draft of the manuscript. FA, DS, JKL, and BR revised and edited the manuscript. BR acquired funding and oversaw the project. All authors contributed to the article and approved the submitted version.

## Funding

This work was supported by the National Institutes of Health (to B.R., R01AI146857-01A1).

## Author Disclaimer

The content is solely the responsibility of the authors and does not necessarily represent the official views of the National Institutes of Health.

## Conflict of Interest

The authors declare that the research was conducted in the absence of any commercial or financial relationships that could be construed as a potential conflict of interest.

## Publisher’s Note

All claims expressed in this article are solely those of the authors and do not necessarily represent those of their affiliated organizations, or those of the publisher, the editors and the reviewers. Any product that may be evaluated in this article, or claim that may be made by its manufacturer, is not guaranteed or endorsed by the publisher.

## Supplementary Material

Supplementary Figure S1 Lung pathology scores and BALF protein concentration in WT and *Rgs10-/-* mice at 3 dpi. Lung histopathological changes were evaluated in WT and *Rgs10-/-* mice (n=6) infected intranasally with 100 PFU of PR8 virus at 3 dpi. Lung histopathological scores were assessed in or around bronchioles (bronchiolitis) **(A)** or alveolar spaces (alveolitis) **(B)**. Data presented in **(A, B)** were analyzed for statistical differences using unpaired t-test between groups. **(C)** BALF protein concentrations were evaluated in uninfected (0 dpi) WT and *Rgs10-/-* mice (n = 5) or WT and *Rgs10-/-* mice (n = 6) infected intranasally with 100 PFU of PR8 virus at 3 dpi. Data were analyzed for statistical differences using an analysis of variance (ANOVA) followed by Tukey *post-hoc* test between groups. Data are presented as mean ± SEM where **p < 0.01; n.s. not significant.

Supplementary Figure S2 Levels of cytokines in BALFs of influenza-infected mice unaffected by *Rgs10* deficiency. The bead-based Bio-Plex assay was used to measure concentrations of the indicated cytokines and chemokines in the BAL supernatants of uninfected (0 dpi) WT and *Rgs10-/-* mice (n=5) or WT and *Rgs10-/-* mice (n=7) infected intranasally with 100 PFU of PR8 virus at 5 dpi. Data were analyzed for statistical differences using an analysis of variance (ANOVA) followed by Tukey *post-hoc* test between groups. Data are presented as mean ± SEM.

Supplementary Figure S3 Flow cytometry gating strategy: myeloid cells. A representative schematic depicting the method for selecting myeloid cell populations present within the BAL of WT and *Rgs10-/-* mice. Single cells negative for zombie aqua fixable viability dye were considered live. The myeloid cell marker CD11b was used to separate myeloid cells from other cell types. The CD11b+ cells were considered the parent cell population for all myeloid cell types measured. The markers used for each cell type are as follows: neutrophils (CD11b^+^, CD115^−^, Ly6G^high^), alveolar macrophages (CD11b^+^, CD115^−^, CD11c^+^, F4/80^+^), dendritic cells (CD11b^+^, CD115^−^, CD11c^+^, F4/80^−^), eosinophils (CD11b^+^, CD115^−^, CD11c^−^, Ly6G^−^, Ly6C^−^, F4/80^+^), monocytes (CD11b^+^, CD115^+^, Ly6G^+^), inflammatory monocytes (CD11b^+^, CD115^+^, Ly6C^high^) and inflammatory macrophages (CD11b^+^, CD115^+^, F4/80^+^, CD11c^−^).

Supplementary Figure S4 Flow cytometry gating strategy: lymphoid cells. A representative schematic depicting the method for selecting lymphoid cell populations present within the BAL of WT and *Rgs10-/-* mice. Single cells negative for zombie aqua fixable viability dye were considered live. CD45+ cells were considered the parent cell population for all cell types measured. The markers used for each cell type are as follows: T cells (CD45^+^, CD3^+^), CD8 T cells (CD45^+^, CD3^+^, CD8^+^, CD4^-^), CD4 T cells (CD45^+^, CD3^+^, CD4^+^, CD8^-^), B cells (CD45^+^, CD3^−^, B220^+^), and natural killer cells (CD45^+^, CD3^−^, NK 1.1^+^).

Supplementary Figure S5 Characterization of leukocytes in uninfected airways of WT and *Rgs10-/-* mice. Indicated myeloid **(A)** and lymphoid **(B)** leukocyte subsets were measured by flow cytometry in BALFs of uninfected WT and *Rgs10-/-* mice (n = 10). Data were analyzed for statistical differences using unpaired t-test between groups. Data are presented as mean ± SEM.

Supplementary Figure S6 No difference in the cellular profiling between infected WT and *Rgs10-/-* at 3 dpi. Phenotypic analysis of myeloid and lymphoid leukocyte subsets was measured by flow cytometry in PR8-infected lung BALF of WT and *Rgs10-/-* mice (n = 9) at 3 dpi. Data were analyzed for statistical differences using unpaired t-test between groups. Data are presented as mean ± SEM.

## Abbreviations

BALF, bronchoalveolar lavage fluid; COX-2, cyclooxygenase-2; dpi, day post-infection; GAP, GTPase-activating protein; GPCR, G protein coupled receptor; KO, gene knockout; IAV, influenza A virus; IL, interleukin; i.n., intranasally; iNOS, inducible nitric oxide synthase; LPS, lipopolysaccharide; MPO, myeloperoxidase; NE, neutrophil elastase; NETs, neutrophil extracellular traps; NF*-*κB, nuclear factor-kappa B; PFU, plaque-forming unit; PGE2, prostaglandin E2; PR8, A/Puerto Rico/8/1934; RGS, regulator of G-protein signaling; RT-PCR, reverse transcription-polymerase chain reaction; TF, transcription factor; TLR, toll-like receptor; TNF-α, tumor necrosis factor-alpha; WT, wild-type.
