# Supplementary material for: RGS10 Reduces Lethal Influenza Infection and Associated Lung Inflammation in Mice
Source: Front Immunol. 2021 Nov 29;12:772288. doi: 10.3389/fimmu.2021.772288 (PMC8667315; doi:10.3389/fimmu.2021.772288)
Supplement: Supplementary Figure S1 — Lung pathology scores and BALF protein concentration in WT and Rgs10-/- mice at 3 dpi. Lung histopathological changes were evaluated in WT and Rgs10-/- mice (n=6) infected intranasally with 100 PFU of PR8 virus at 3 dpi. Lung histopathological scores were assessed in or around bronchioles (bronchiolitis) (A) or alveolar spaces (alveolitis) (B). Data presented in (A, B) were analyzed for statistical differences using unpaired t-test between groups. (C) BALF protein concentrations were evaluated in uninfected (0 dpi) WT and Rgs10-/- mice (n = 5) or WT and Rgs10-/- mice (n = 6) infected intranasally with 100 PFU of PR8 virus at 3 dpi. Data were analyzed for statistical differences using an analysis of variance (ANOVA) followed by Tukey post-hoc test between groups. Data are presented as mean ± SEM where **p < 0.01; n.s. not significant. [file DataSheet_1.pdf]

RGS10 reduces lethal influenza infection and associated lung inflammation in mice

Faris Almutairi, Demba Sarr, Samantha L. Tucker, Kayla Fantone, Jae-Kyung Lee and Balázs Rada

Supplementary figures

Fig. S1

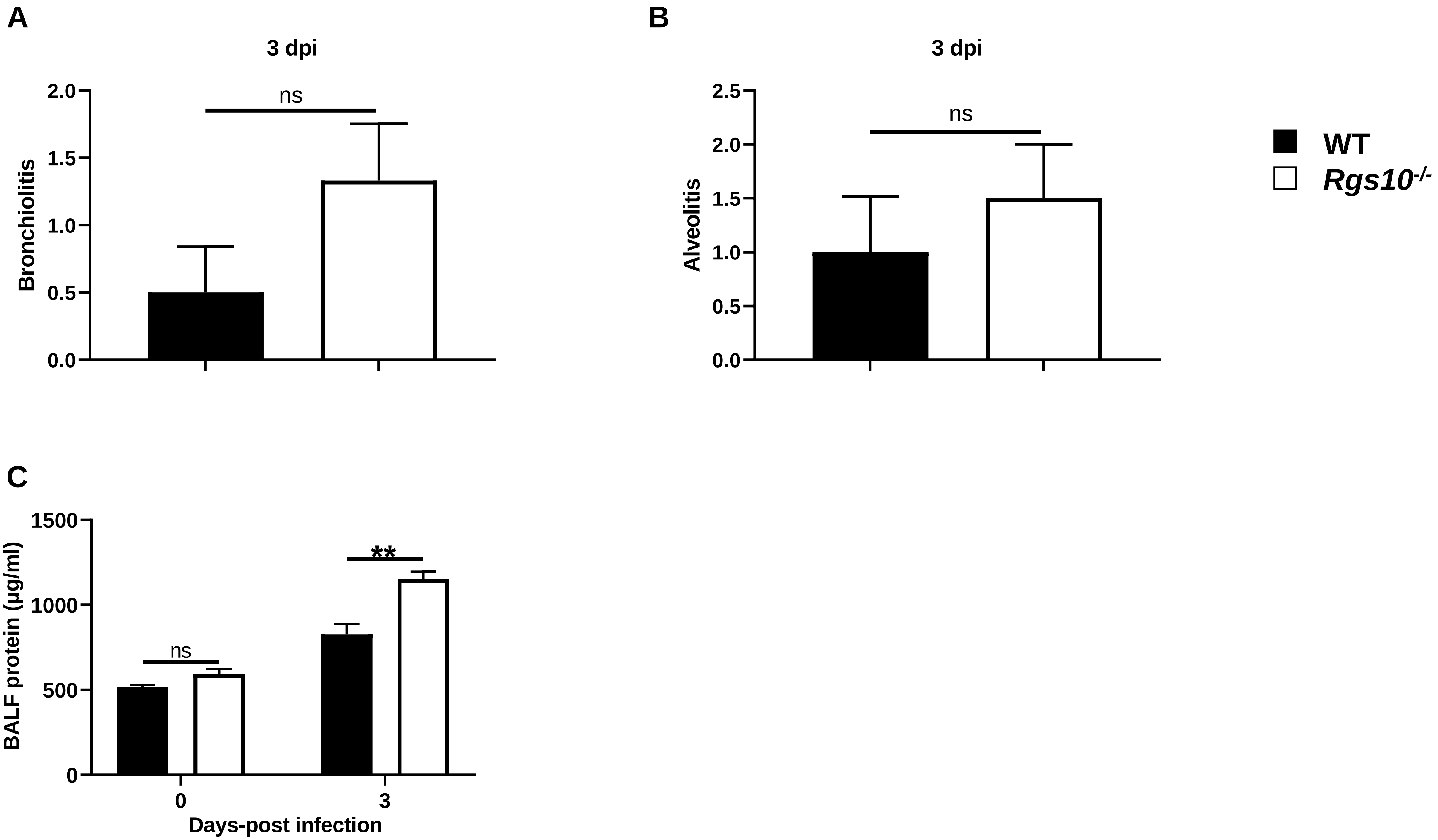
A
3 dpi
Bronchiolitis
2.0
1.5
1.0
0.5
0.0
ns
B
3 dpi
Alveolitis
2.5
2.0
1.5
1.0
0.5
0.0
ns
WT
Rgs10-/-
C
BALF protein (µg/ml)
1500
1000
500
0
ns
**
0
3
Days-post infection

Fig. S2

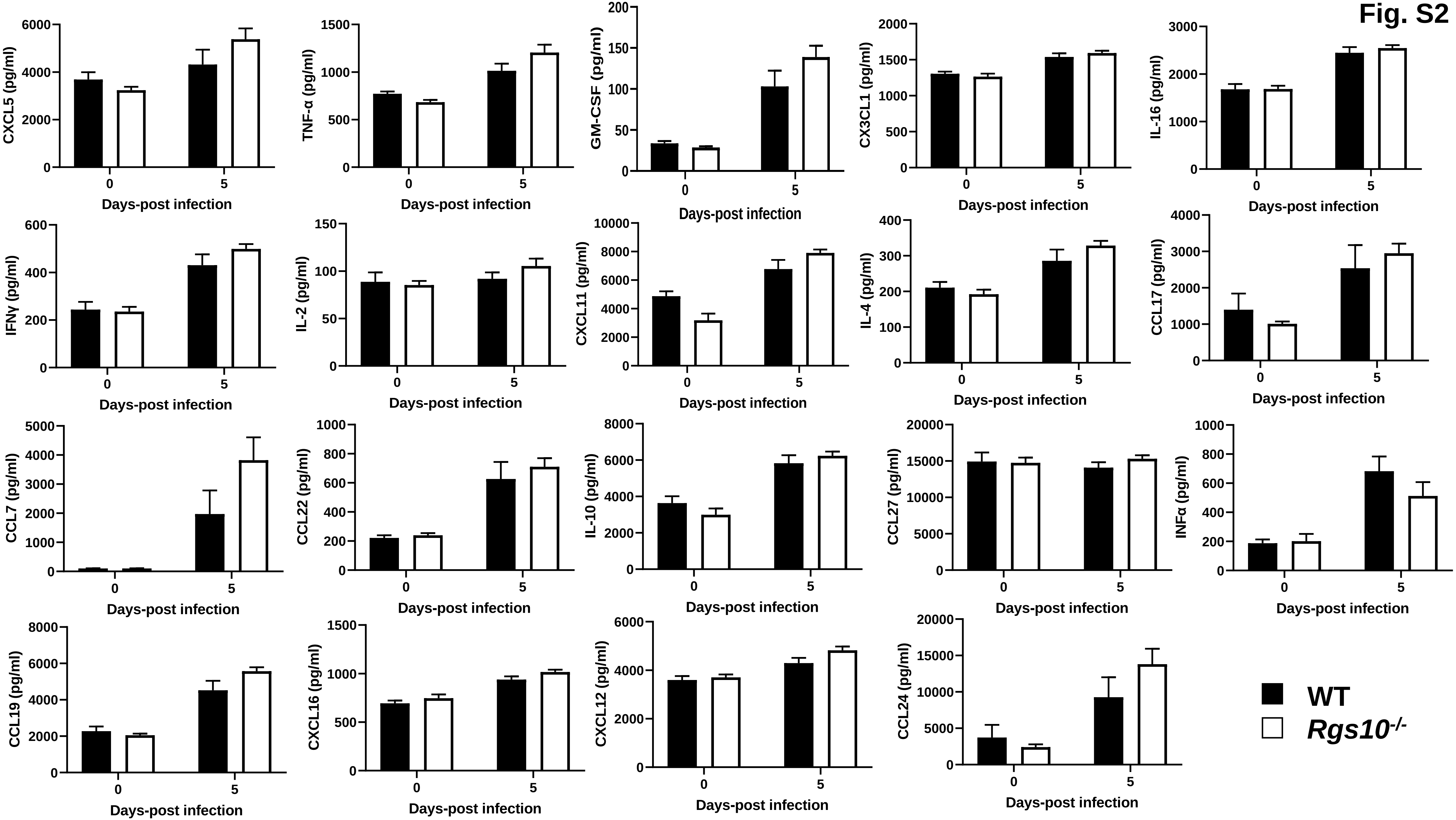
CXCL5 (pg/ml)
TNF-α (pg/ml)
GM-CSF (pg/ml)
CX3CL1 (pg/ml)
IL-16 (pg/ml)
IFNγ (pg/ml)
IL-2 (pg/ml)
CXCL11 (pg/ml)
IL-4 (pg/ml)
CCL17 (pg/ml)
CCL7 (pg/ml)
CCL22 (pg/ml)
IL-10 (pg/ml)
CCL27 (pg/ml)
INFα (pg/ml)
CCL19 (pg/ml)
CXCL16 (pg/ml)
CXCL12 (pg/ml)
CCL24 (pg/ml)
Days-post infection
WT
Rgs10-/-

Fig. S3

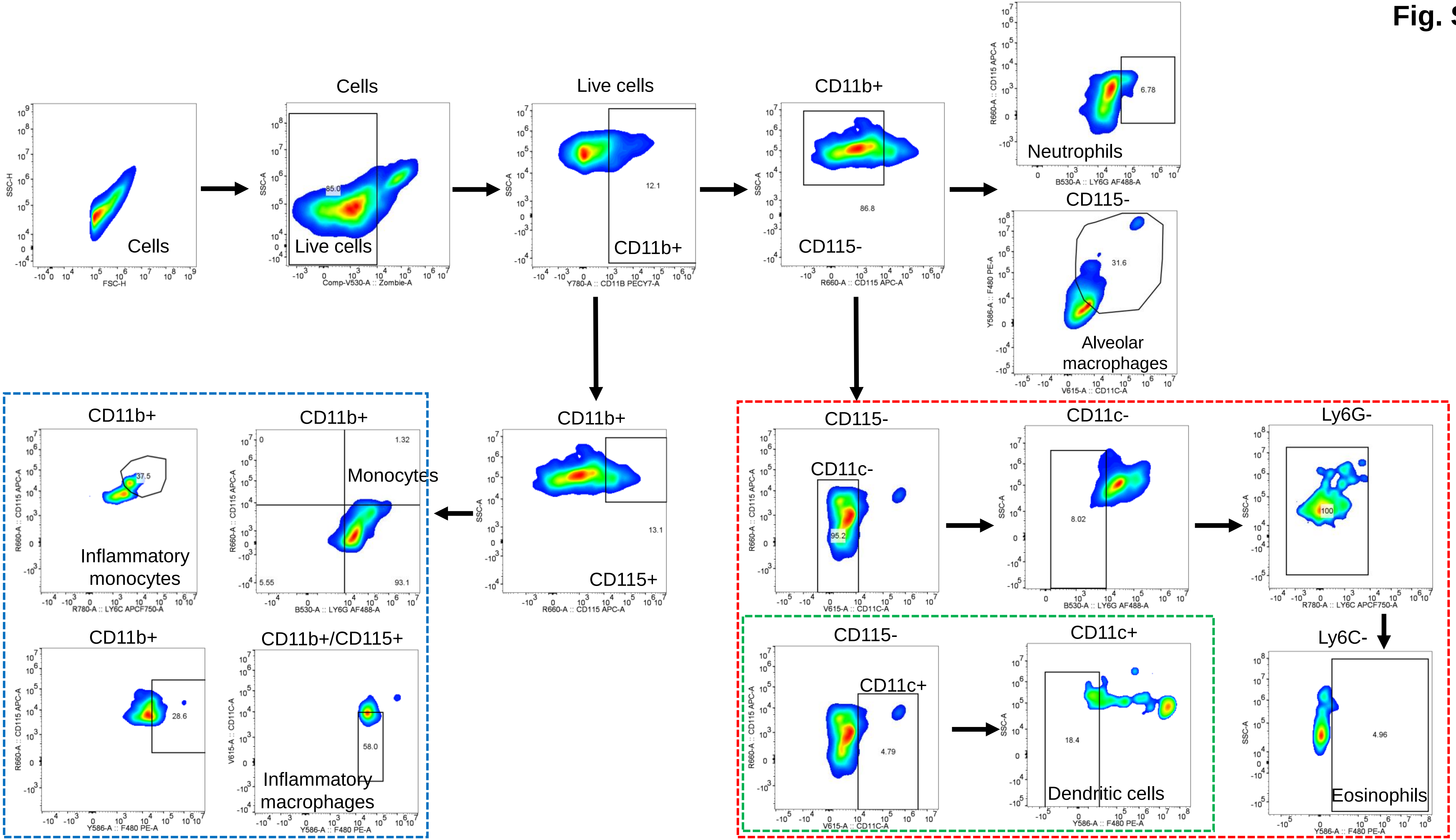

Cells
Live cells
CD11b+
Cells
Live cells
85.0
CD11b+
12.1
CD115-
86.8
Neutrophils
6.78
CD115-
31.6
Alveolar macrophages
CD11b+
Inflammatory monocytes
37.5
CD11b+
Monocytes
1.32
5.55
93.1
0
CD11b+
CD115+
13.1
CD115-
CD11c-
95.2
CD11c-
8.02
Ly6G-
100
CD11b+
28.6
CD11b+/CD115+
58.0
Inflammatory macrophages
CD115-
CD11c+
4.79
CD11c+
18.4
Dendritic cells
Ly6C-
4.96
Eosinophils
FSC-H
SSC-H
SSC-A
Comp-V530-A :: Zombie-A
Y780-A :: CD11B PECY7-A
R660-A :: CD115 APC-A
B530-A :: LY6G AF488-A
Y586-A :: F480 PE-A
V615-A :: CD11C-A
R780-A :: LY6C APCF750-A
V615-A :: CD11C-A

Fig. S4

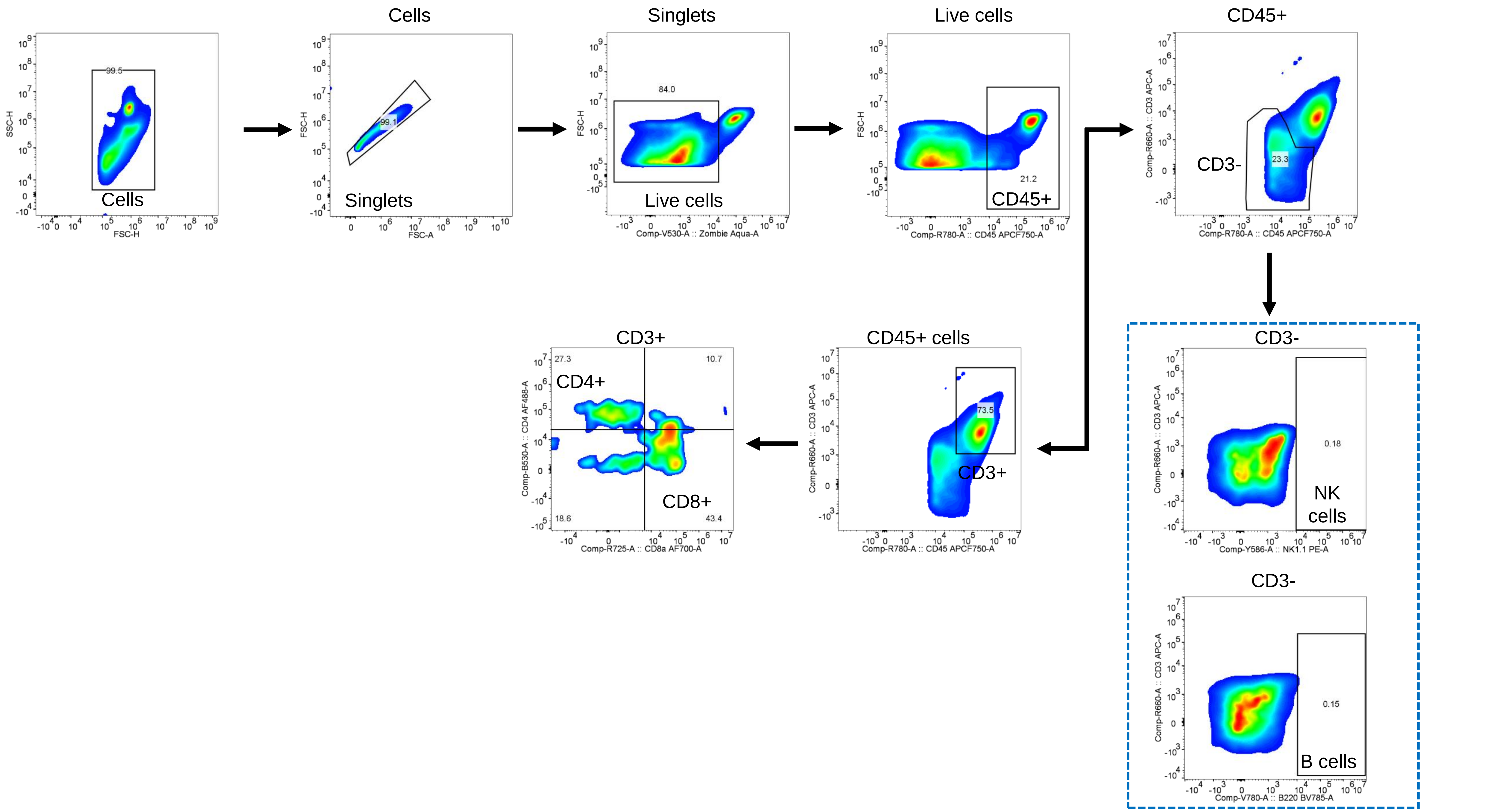
Cells
Singlets
Live cells
CD45+
99.5
Cells
SSC-H
FSC-H
99.1
Singlets
FSC-H
FSC-A
84.0
Live cells
FSC-H
Comp-V530-A :: Zombie Aqua-A
21.2
CD45+
FSC-H
Comp-R780-A :: CD45 APCF750-A
CD3-
23.3
Comp-R660-A :: CD3 APC-A
Comp-R780-A :: CD45 APCF750-A
CD3+
27.3
10.7
CD4+
CD8+
18.6
43.4
Comp-B530-A :: CD4 AF488-A
Comp-R725-A :: CD8a AF700-A
CD45+ cells
73.5
CD3+
Comp-R660-A :: CD3 APC-A
Comp-R780-A :: CD45 APCF750-A
CD3-
0.18
NK cells
Comp-R660-A :: CD3 APC-A
Comp-Y586-A :: NK1.1 PE-A
CD3-
0.15
B cells
Comp-R660-A :: CD3 APC-A
Comp-V780-A :: B220 BV785-A

Fig. S5

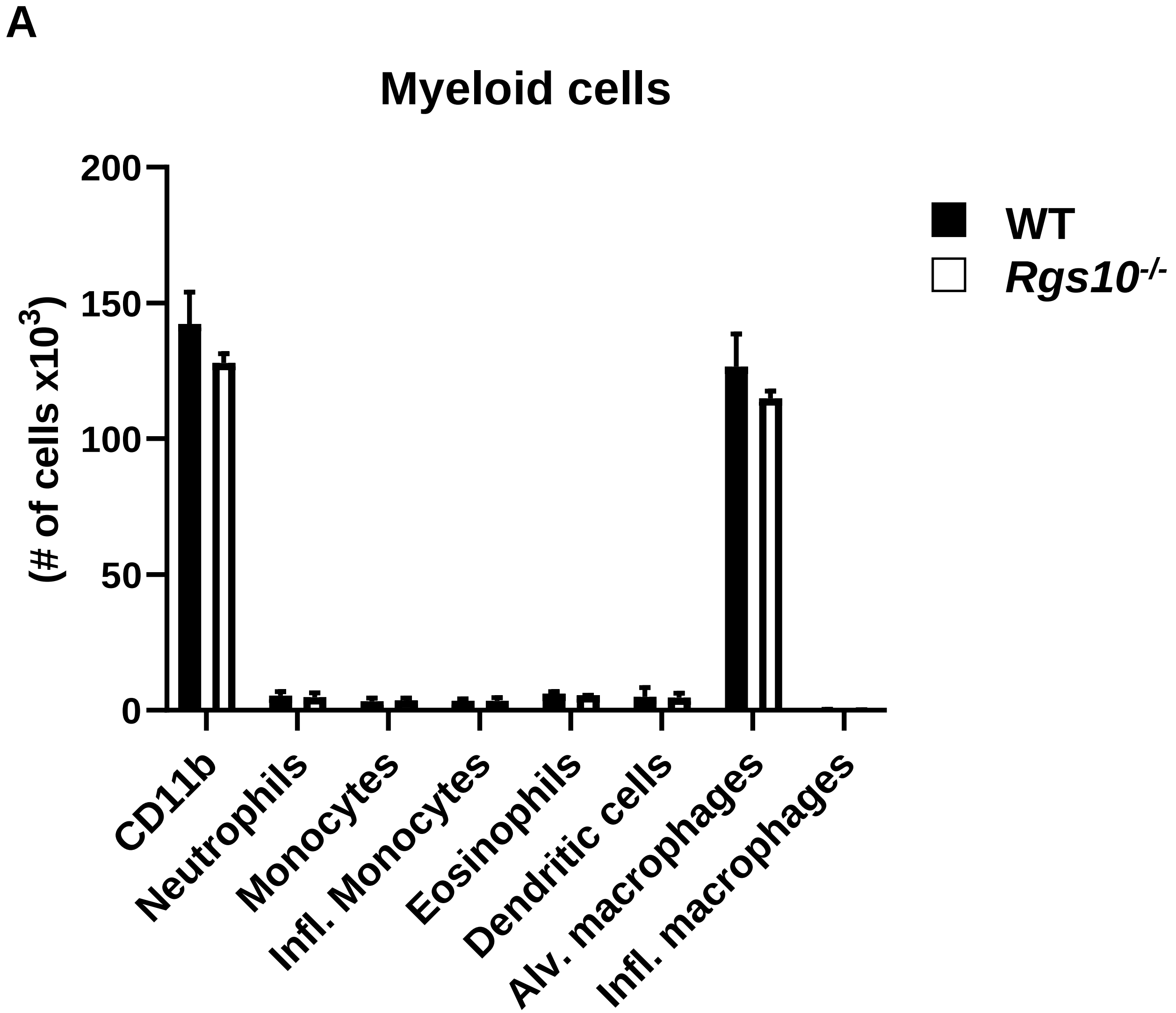
A
Myeloid cells
200
150
100
50
0
(# of cells x10^3)
WT
Rgs10-/-
CD11b
Neutrophils
Monocytes
Infl. Monocytes
Eosinophils
Dendritic cells
Alv. macrophages
Infl. macrophages

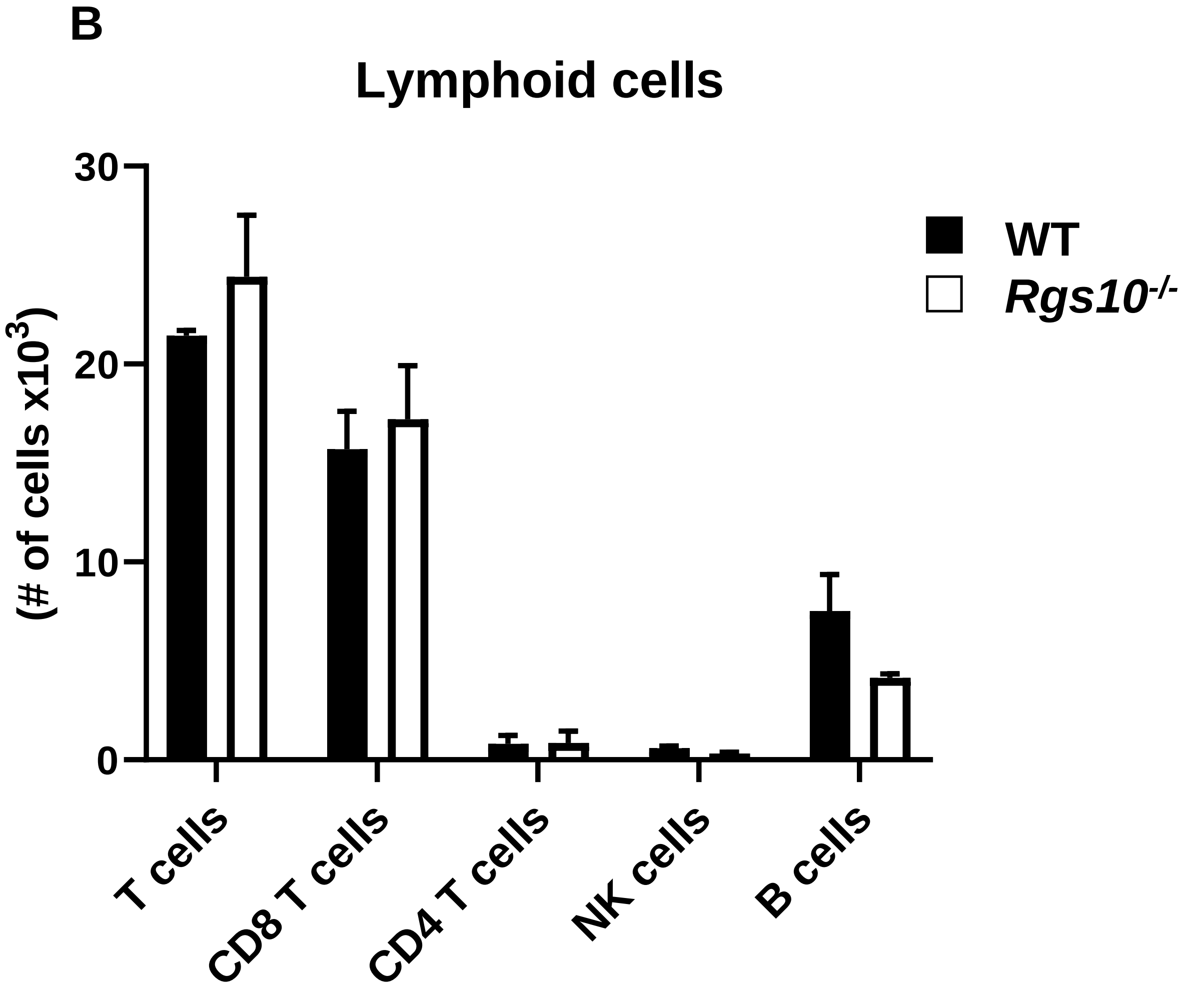
B
Lymphoid cells
30
20
10
0
(# of cells x10^3)
WT
Rgs10-/-
T cells
CD8 T cells
CD4 T cells
NK cells
B cells

Fig. S6

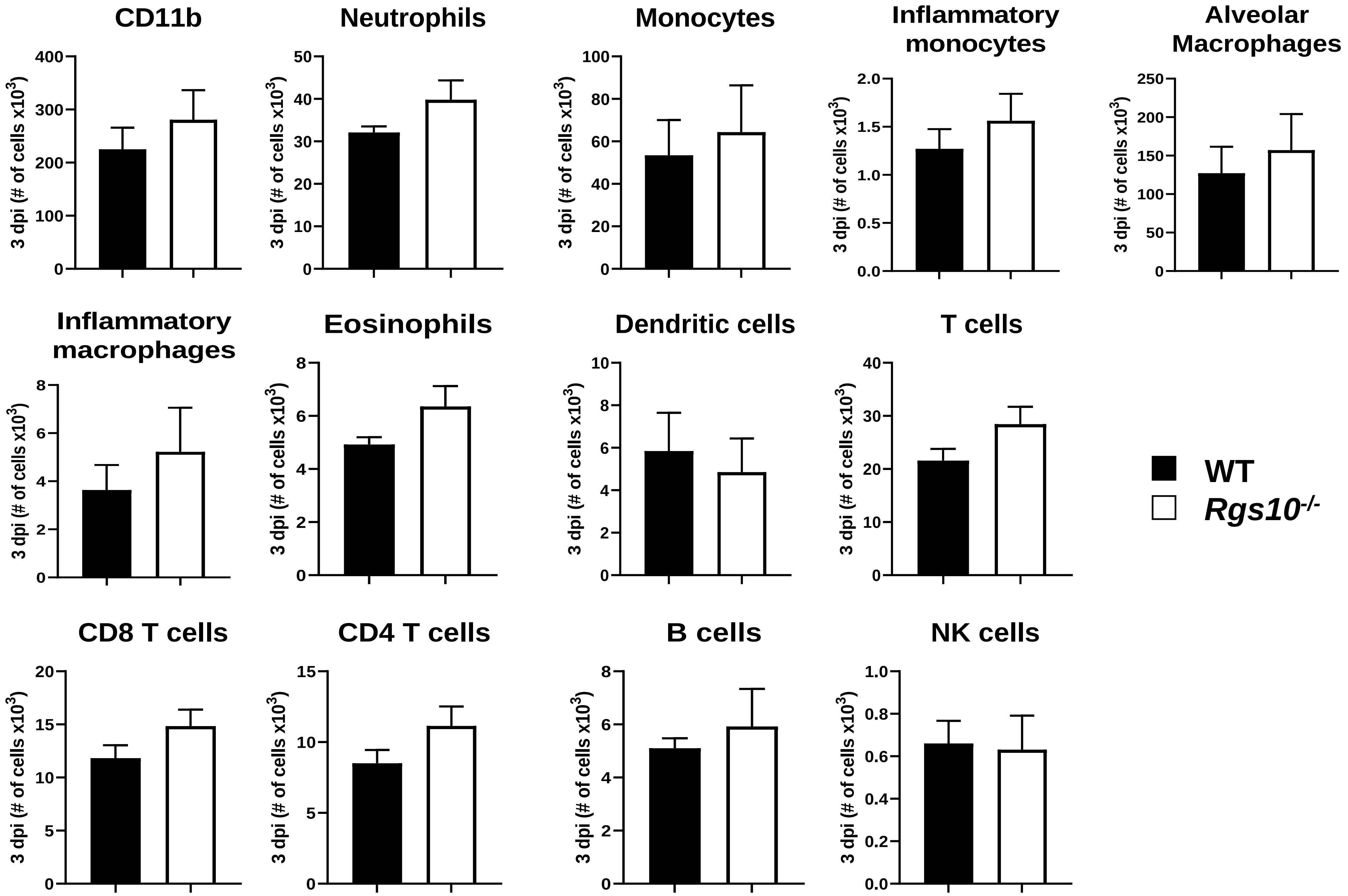
CD11b
Neutrophils
Monocytes
Inflammatory monocytes
Alveolar Macrophages
Inflammatory macrophages
Eosinophils
Dendritic cells
T cells
CD8 T cells
CD4 T cells
B cells
NK cells
3 dpi (# of cells x10³)
WT
Rgs10-/-
